# Predictive role of neutrophil percentage-to-albumin ratio in acute fulminant myocarditis patients receiving extracorporeal membrane oxygenation

**DOI:** 10.1007/s12519-025-00940-4

**Published:** 2025-07-17

**Authors:** Jing-Jing Zhou, Jun-Jie Shao, Shuai Yue, Hao-Jie Yan, Hai-Ming Wang, Min Jiang, Shu-Jin Shi, Shuai Xu, Jun-Jie Su, Fan Han, Xiao-Yang Hong, Ran Zhang

**Affiliations:** 1 Department of Cardiovascular Medicine, Chinese PLA General Hospital, Beijing, 100853 China; 2 Graduate School of Chinese PLA Medical School, Beijing, 100853 China; 3 Department of Pulmonary and Critical Care Medicine, Chinese PLA General Hospital, Beijing, 100853 China; 4 Division of Pediatric Intensive Care, Department of Pediatrics, the Seventh Medical Center of Chinese PLA General Hospital, 5 South Gate Warehouse, Dongsi Liu Tiao, Beijing, China; 5 Department of Cardiovascular Medicine, Chinese PLA General Hospital & Chinese PLA Medical School, 28 Fuxing Road, Beijing, 100853 China

**Keywords:** Acute fulminant myocarditis, Inflammation, Neutrophil percentage-to-albumin ratio, Nomogram, Pediatrics, Venoarterial extracorporeal membrane oxygenation

## Abstract

**Background:**

Venoarterial extracorporeal membrane oxygenation induces an inflammatory response upon initiation. The neutrophil percentage-to-albumin ratio is a promising biomarker for predicting mortality in patients with systemic inflammation. This study aimed to investigate the association between neutrophil percentage-to-albumin ratio and in-hospital mortality in pediatric patients with acute fulminant myocarditis undergoing extracorporeal membrane oxygenation and to develop a PEACE model for predicting mortality in these patients.

**Methods:**

This retrospective study included pediatric patients diagnosed with acute fulminant myocarditis who underwent venoarterial extracorporeal membrane oxygenation between July 2015 and August 2022. Multivariable logistic regression analysis was used to investigate the independent association between the neutrophil percentage-to-albumin ratio and the risk of in-hospital mortality. In addition, we utilized least absolute shrinkage and selection operator regression to select predictive factors, ultimately developing a nomogram to predict outcomes in pediatric patients receiving venoarterial extracorporeal membrane oxygenation.

**Results:**

A total of 125 patients eligible for analysis were included in this study, with an in-hospital mortality rate of 28.8%. Multivariable logistic regression revealed that the neutrophil percentage-to-albumin ratio was an independent risk factor for in-hospital mortality in venoarterial extracorporeal membrane oxygenation patients. Restricted cubic splines revealed a positive association between the two (*P*_nonlinearity_ = 0.84). Least absolute shrinkage and selection operator regression and backward stepwise logistic regression identified age, cardiopulmonary resuscitation, lactate levels, and the neutrophil percentage-to-albumin ratio as key predictive factors. Using these factors, a nomogram (PEACE model) was developed to predict in-hospital mortality in venoarterial extracorporeal membrane oxygenation patients. The area under the receiver operating characteristic curve was 0.83 [95% confidence interval (CI), 0.74–0.92], with the inclusion of the neutrophil percentage-to-albumin ratio significantly enhancing the model’s predictive accuracy.

**Conclusions:**

The neutrophil percentage-to-albumin ratio may serve as a potential predictor for venoarterial extracorporeal membrane oxygenation in-hospital mortality in pediatric patients with acute fulminant myocarditis, suggesting that inflammatory responses are associated with patient prognosis. The PEACE model is superior in predicting the prognosis of pediatric patients supported by venoarterial extracorporeal membrane oxygenation, and can help in clinical decision making.

**Graphical abstract:**

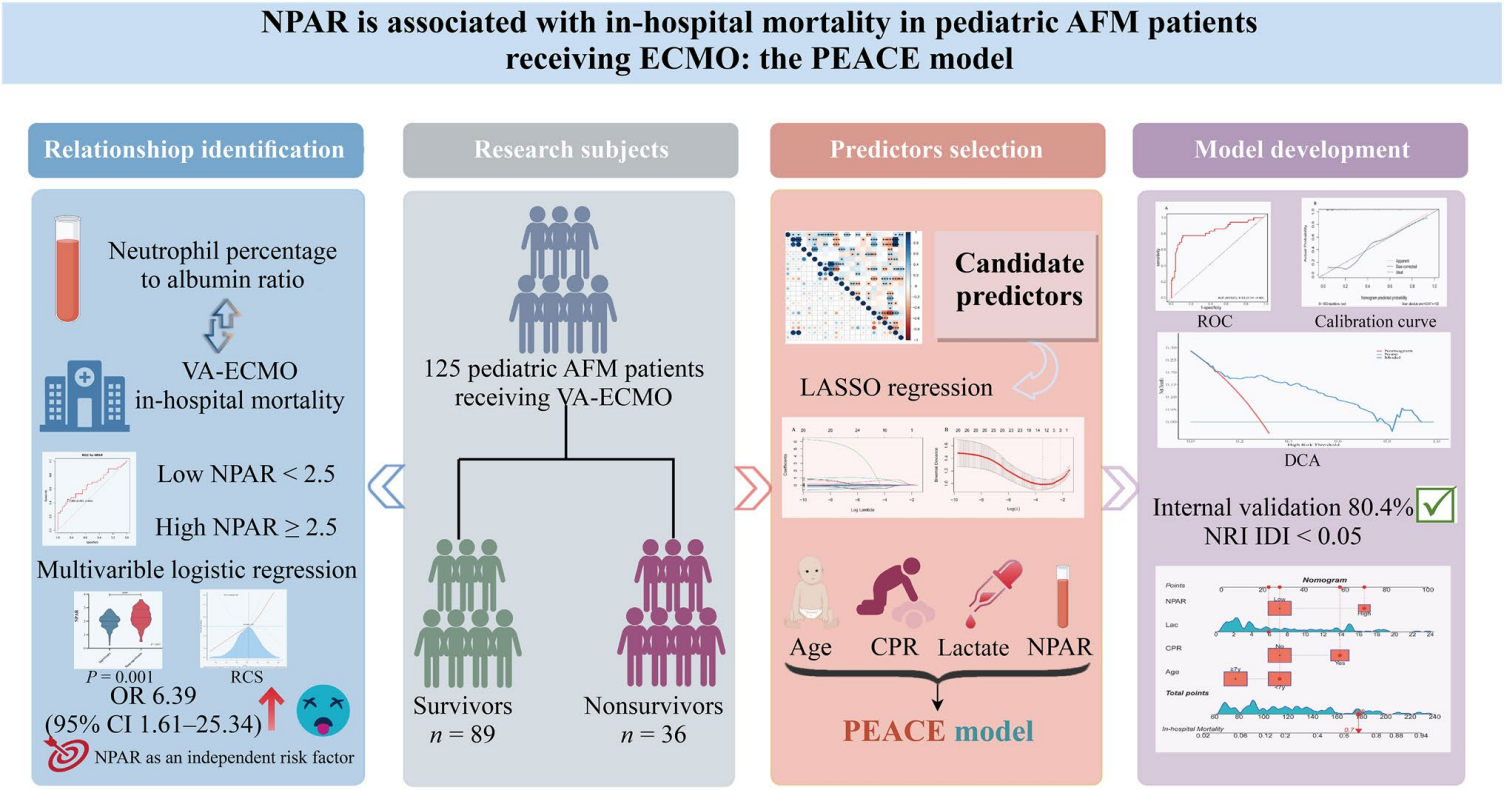

**Supplementary Information:**

The online version contains supplementary material available at 10.1007/s12519-025-00940-4.

## Introduction

Acute fulminant myocarditis (AFM) is a complex clinical condition characterized by the rapid onset of ventricular arrhythmias and cardiogenic shock. Accurate and timely diagnosis of AFM is challenging because of its rapid progression and severe manifestations [1–3]. The mortality rate for newborns and children diagnosed with AFM remains alarmingly high [4]. When conventional vasoactive drugs are insufficient to maintain blood pressure and organ perfusion, veno-arterial extracorporeal membrane oxygenation (VA-ECMO) has emerged as a valuable support to improve survival [5–8]. VA-ECMO is a temporary mechanical support technology that draws blood from large veins, oxygenates it, and returns it to circulation through large arteries, providing critical support to the heart [9-10]. In addition to the systemic inflammatory response to the disease itself, this process of the patient's blood coming into contact with the nonendothelial surface of the extracorporeal circuit could trigger an inflammatory response and activate the innate immune system [11].

Previous studies have shown that there is a strong association between systemic inflammation and mortality in patients undergoing VA-ECMO [12, 13]. In this context, the peripheral blood neutrophil count and percentage serve as readily available and inexpensive indicators of systemic inflammation. Serum albumin, which reflects nutritional status and has antioxidant and anti-inflammatory properties, can provide valuable insight into the inflammatory response [14, 15]. The neutrophil percentage-to-albumin ratio (NPAR), a combination of these two biomarkers, has been extensively studied in conditions, such as acute myocardial infarction, cardiogenic shock, atrial fibrillation [16], encephalitis [17], acute kidney injury, and liver disease [18]. Research suggests that the NPAR is a potential inflammatory indicator for predicting patient mortality [16]. However, the relationship between the NPAR and mortality in pediatric patients with AFM receiving VA-ECMO support remains to be fully elucidated. The primary objective of this retrospective study was to evaluate the prognostic significance of the NPAR in pediatric patients with AFM receiving VA-ECMO support and to develop a clinical nomogram model (PEACE model) to predict in-hospital mortality.

## Methods

### Study design and population

We consecutively enrolled all pediatric patients with AFM who underwent VA-ECMO in eight tertiary pediatric intensive care units in China between July 2015 and August 2022. The diagnosis of acute myocarditis was made by experienced pediatricians on the basis of the following clinical criteria: (1) a recent history of viral infection with symptoms, such as abdominal pain, vomiting, palpitations, fatigue and fever; (2) electrocardiographic abnormalities [including atrioventricular block, ST-T changes, widening of the Q, R, and S peaks (QRS) complex or PR prolongation] together with elevated cardiac enzymes; (3) signs of acute heart failure and significantly reduced left ventricular ejection fraction (LVEF); and (4) no prior history of cardiac disease [19]. Acute myocarditis may be classified as AFM if it presents suddenly and progresses rapidly to severe heart failure, hypotension or cardiogenic shock requiring the use of positive inotropes, vasopressors or mechanical circulatory support. Endomyocardial biopsies and magnetic resonance imaging are often not performed in children with AFM because of their critical condition [20]. VA-ECMO is indicated for cardiogenic shock, which is defined as refractory to standard medical therapies, persistent systemic systolic pressure less than 50 mmHg, urine output < 1 mL/kg/hour, lactic acidosis, central venous oxygen saturation (SVO_2_) < 60%, and altered mental status due to low cardiac output [21]. If patients underwent more than two VA-ECMO runs during the study period, we included data from only the first run. Patients with incomplete information, missing survival status, or ECMO duration of less than 24 hours were excluded from the analysis. This retrospective study was approved by the institutional ethics committees of the Seventh Medical Center of Chinese PLA General Hospital (approval number 2018–15; granted on March 5, 2018), and the requirement for written informed consent was waived owing to the use of deidentified data. The PEACE model was reported in compliance with the Transparent Reporting of a Multivariable Prediction Model for Individual Prognosis or Diagnosis (TRIPOD) statement [22] (Supplemental Text 1). The overall study design is summarized in the graphical abstract.

### Veno-arterial extracorporeal membrane oxygenation implantation and management

Implantation of VA-ECMO was always performed by a trained ECMO team. Cannulation is typically performed via a percutaneous approach with the Seldinger technique, a surgical cut-down method, or a combination of both. Cannula size was generally selected on the basis of the child's weight, cannulation site, and vessel diameter. Prior to cannulation, peripheral vessel diameters were assessed via vascular ultrasound. In children weighing less than 15 kg, the internal jugular vein or the common carotid artery are preferred sites for cannulation, as the smaller femoral vessels pose a challenge. This approach ensures optimal ECMO flow through the larger vessels of the upper body and reduces the risk of potential neurological complications. For children weighing more than 30 kg, the femoral vein and artery could be used. When the femoral artery was used, an additional catheter was placed distal to the cannulation site to prevent distal ischemia. Cannula sizes of 14F and smaller are typically used for cervical vessels; those of 15F and larger are commonly used for femoral vessels, and larger sizes are occasionally used for the neck in special cases. The initial ECMO blood flow was set at 100–150 mL/kg·minute for neonates and infants and 80–120 mL/kg·minute for children [21, 23]. The pump speed was adjusted to achieve a blood lactate level of less than 1.5 mmol/L or a systemic SVO_2_ greater than 70%. In addition, the aim was to maintain a minimum mean arterial pressure (MAP) of 40–50 mmHg for neonates and 50–60 mmHg for children [21]. The hematocrit (Hct) was maintained between 0.35 and 0.40 to optimize oxygen delivery with the lowest perfusion flow [24]. The activated clotting time (ACT) was maintained at a range of 180–200 seconds by administering heparin intravenously, as long as there was no uncontrolled bleeding [25, 26]. In addition, left heart decompression was performed in cases, where cardiac function was severely inadequate, resulting in ventricular congestion and pulmonary edema. The ECMO weaning trial was initiated when the intensity of ECMO support was less than 30% of the patient’s heart or lung function [27].

### Data collection and the association of NPAR with the outcome

Demographic information, routine laboratory tests, and clinical characteristics were retrieved from the electronic medical records system, with values recorded 12 hours after VA-ECMO initiation. Variables such as the neutrophil-to-lymphocyte ratio (NLR) and NPAR were defined. In addition, data on the duration of VA-ECMO, whether cardiopulmonary resuscitation (CPR) was performed, and discharge status were documented for each patient. Detailed information on the variables collected and defined can be found in Supplementary Table 1. Two experienced investigators independently collected retrospective data via a standardized form. Discrepancies were resolved through discussion and consensus, with a third senior investigator providing adjudication when necessary.

The primary outcome of the study was in-hospital mortality, which was defined as all-cause death prior to hospital discharge. To investigate the association between the NPAR and in-hospital mortality, we determined the optimal NPAR cutoff using a receiver operating characteristic (ROC) curve and standardized (*Z* score) the NPAR to fit binary logistic regression models, accompanied by odds ratios (ORs) and 95% confidence intervals (CIs). In the multivariable logistic regression analysis of the NPAR, adjustments were made for variables with *P* values < 0.01 from the univariable logistic analysis, as well as for clinical factors (age, sex, weight, duration of ECMO) and independent risk factors identified in previous studies, such as the NLR [13]. Of note, some variables (neutrophil count, lymphocyte count, neutrophil percentage and albumin) used to calculate alternative variables (such as NLR and NPAR) were not included as covariates in the analysis to prevent over-adjustment. Restricted cubic splines (RCSs), which are based on multivariable logistic regression models, were applied to assess the dose‒response relationship between the NPAR and in-hospital mortality [28]. Subgroup analyses were conducted to explore potential interactions between the NPAR and various subgroup characteristics.

### Predictor selection and model construction

A heatmap was generated to illustrate the potential correlations between candidate variables. To refine variable selection and minimize the risk of collinearity and overfitting, least absolute shrinkage and selection operator (LASSO) regression was applied, accompanied by tenfold cross-validation for internal validation. Variables with nonzero coefficients identified by LASSO regression were included in the model. In addition, the clinical variables (such as age, sex and weight) associated with mortality in previous studies were forced into the multivariable logistic regression analysis via the backward stepwise method to identify predictive factors. The model was then visualized via a nomogram to facilitate the prediction of patient outcomes. On the basis of scores calculated from the nomogram, patients were stratified into low-, intermediate-, and high-risk groups, with Bonferroni correction for multiple comparisons.

### Model assessment and internal validation

The performance of the model was assessed by discrimination via the area under the curve (AUC) from the ROC curve. The accuracy was assessed via calibration via a calibration curve and the Hosmer–Lemeshow test, with associated *P* values. Decision curve analysis (DCA) was applied to estimate the net clinical benefit. In addition, internal validation was conducted by bootstrapping with 1000 resamples to assess the predictive performance of the model. To further assess the critical role of the NPAR in the nomogram model, we calculated the net reclassification improvement (NRI) and integrated discrimination improvement (IDI) to compare the model with and without the NPAR.

### Statistical analysis

Patient characteristics are summarized as counts and proportions for categorical variables and as the means ± standard deviations (SDs) or medians (interquartile ranges, IQRs) for continuous variables. To assess group differences, categorical variables were compared via the chi-square test or Fisher's exact test, whereas continuous variables were analyzed via Student's *t* test or the Mann‒Whitney *U* test, respectively. To address potential bias due to significant missing data, variables with more than 20% missing values were excluded from further analysis. Multivariable imputation was applied to variables with less than 20% missing data (Supplementary Fig. 1).

All the statistical analyses were performed with RStudio software (version 4.4.1). The interactive web-based dynamic nomogram application was developed via Shiny (version 2.3.0). A *P* value < 0.05 was considered statistically significant.

## Results

### Baseline characteristics of the study population

**Fig. 1:**
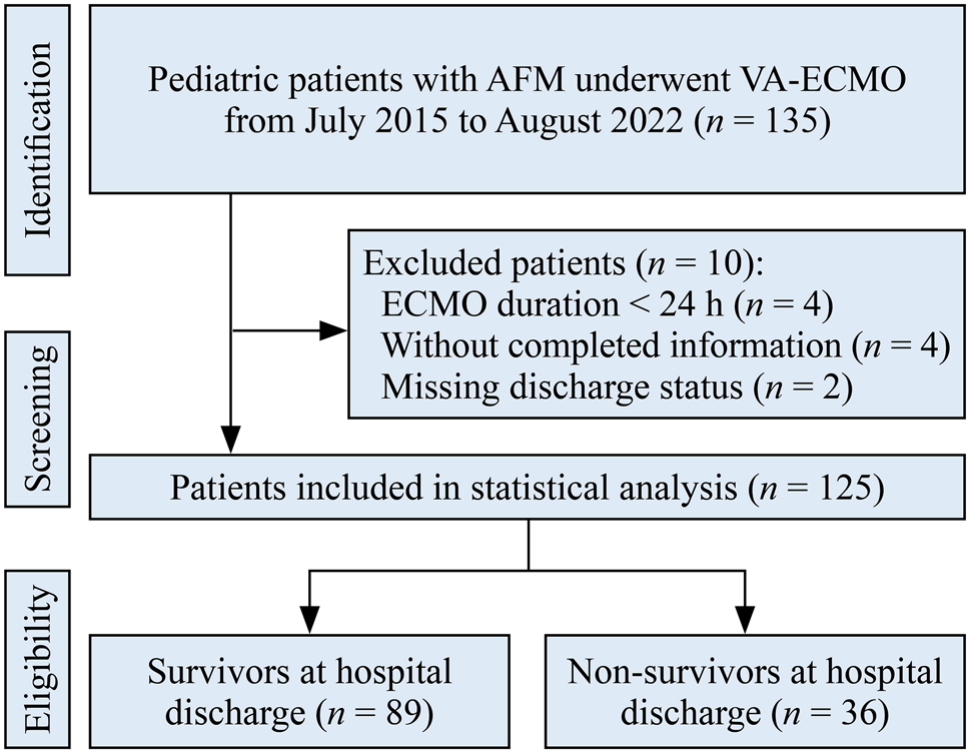
Flowchart of the study population selection process. *AFM* indicates acute fulminant myocarditis, *ECMO* extracorporeal membrane oxygenation, *VA-ECMO* veno-arterial extracorporeal membrane oxygenation

A total of 135 pediatric patients with AFM who were receiving VA-ECMO were initially screened for eligibility. After 10 patients were excluded, 125 patients were included in the analysis (Fig. 1). The median age of the participants was 7.0 years (IQR 4.17–10.0), and the median weight was 23.1 kg (IQR 15.78–36.0), with 47.2% being male. The median duration of VA-ECMO support was 120.17 hours (IQR 92.0–168.0), and the median length of hospital stay was 22.76 ± 14.12 days. No significant differences were observed between survivors and nonsurvivors in terms of age, weight, sex, or duration of VA-ECMO. However, the length of hospital stay was significantly longer in the survivor group than in the nonsurvivor group (28.47 days vs. 8.63 days, *P* < 0.001). Table 1 summarizes the baseline characteristics of both groups.

**Table 1:** Baseline characteristics of study population

Variables	Total	Survivors	Non-survivors	*P* value
Number (%)	125 (100)	89 (71.20)	36 (28.80)	
Demographics				
Age, y	7.00 (4.17, 10.00)	7.33 (5.00, 10.00)	6.00 (2.90, 10.14)	0.212
Weight, kg	23.10 (15.78, 36.00)	23.10 (17.00, 37.00)	23.25 (14.30, 34.25)	0.313
Gender, *n* (%)				
Male	59 (47.20)	41 (46.10)	18 (50.00)	0.841
Clinical characteristics				
CPR, *n* (%)	48 (38.40)	24 (27.00)	24 (66.60)	< 0.001
ECMO duration, h	120.17 (92.00, 168.00)	127.13 (106.50, 162.00)	92.75 (32.62, 217.88)	0.084
LOHS, d	22.76 ± 14.12	28.47 ± 11.70	8.63 ± 8.58	< 0.001
Blood pressure, mmHg				
Systolic	76.00 (60.00, 95.00)	80.00 (66.00, 96.00)	67.00 (50.50, 76.25)	0.004
Diastolic	47.00 (35.00, 60.00)	52.00 (38.00, 60.00)	39.00 (28.75, 54.25)	0.007
Mean arterial pressure	56.67 (44.33, 70.33)	60.00 (48.67, 73.33)	50.33 (36.67, 60.17)	0.004
Laboratory tests				
Hemoglobin, g/L	113.00 (98.00, 124.00)	116.00 (104.00, 124.00)	101.00 (92.50, 120.00)	0.039
Red blood distribution width, %	14.00 (12.90, 35.20)	13.70 (12.70, 32.16)	15.65 (13.50, 43.25)	0.004
Hematocrit, %	32.57 ± 5.53	33.56 ± 5.30	30.14 ± 5.41	0.002
White blood cell, × 10^9^/L	11.29 (8.06, 15.33)	10.39 (7.57, 13.45)	13.57 (9.83, 17.25)	0.013
Neutrophil count, × 10^9^/L	7.99 (5.11, 11.60)	7.69 (4.98, 10.32)	9.08 (5.82, 12.87)	0.100
Neutrophil percentage, %	74.72 (64.00, 83.30)	74.72 (65.60, 82.50)	75.05 (61.64, 84.33)	0.857
Lymphocyte count, × 10^9^/L	1.74 (1.03, 2.80)	1.46 (0.92, 2.52)	2.50 (1.32, 4.14)	0.006
Neutrophil–lymphocyte ratio	4.44 (2.34, 8.45)	4.44 (2.60, 8.26)	4.52 (1.87, 9.36)	0.484
Platelet count, × 10^9^/L	205.00 (126.00, 280.00)	217.00 (164.00, 281.00)	133.50 (95.25, 235.45)	0.010
C-reactive protein, mg/L	5.68 (2.73, 13.00)	6.00 (3.00, 13.58)	5.06 (1.63, 10.25)	0.262
Albumin, g/L	35.50 (32.70, 39.46)	36.17 (33.80, 40.10)	33.33 (26.82, 35.78)	< 0.001
Creatinine, μmol/L	66.00 (48.00, 105.50)	59.00 (47.40, 89.40)	91.34 (67.45, 156.50)	< 0.001
Cardiac troponin I, ng/mL	4.26 (1.55, 10.05)	4.26 (1.47, 9.22)	4.64 (2.12, 13.42)	0.394
Brain natriuretic peptide, pg/mL	15,000.00 (5542.00, 30,790.20)	13,371.00 (5113.00, 27,880.20)	15,661.40 (12,415.85, 34,050.65)	0.137
pH	7.33 (7.20, 7.43)	7.36 (7.23, 7.43)	7.28 (7.10, 7.40)	0.021
PaO_2_, mmHg	91.50 (51.20, 168.00)	108.00 (60.90, 184.10)	68.30 (37.18, 102.01)	0.017
PaCO_2_, mmHg	34.00 (28.20, 42.70)	33.00 (28.00, 40.00)	38.15 (30.86, 50.28)	0.054
Lactate, mmol/L	4.90 (2.40, 11.60)	3.70 (2.00, 7.30)	11.90 (7.25, 15.14)	< 0.001
NPAR, mean ± SD	2.05 ± 0.58	1.95 ± 0.51	2.29 ± 0.68	0.010

Data are presented as counts and proportions for categorical variables and as mean ± standard deviation or median (interquartile range) for continuous variables

*CPR* cardiopulmonary resuscitation, *ECMO* extracorporeal membrane oxygenation, *LOHS* length of hospital stay, *NPAR* neutrophil percentage-to-albumin ratio, *PaO*_*2*_ partial pressure of oxygen, *PaCO*_*2*_ partial pressure of carbon dioxide, *SD* standard deviation

### Association between the neutrophil percentage-to-albumin ratio and the risk of in-hospital mortality

To investigate the potential association between the NPAR and in-hospital mortality, we conducted multiple models via binary logistic regression analysis. The optimal cutoff value for the NPAR, identified by ROC analysis, was 2.5 (Supplementary Fig. 2). Patients were subsequently divided into two groups: the low-NPAR group (NPAR < 2.5) and the high-NPAR group (NPAR ≥ 2.5), comprising 78.4% (98/125) and 21.6% (27/125) of the cohort, respectively. The baseline characteristics of these groups are summarized in Supplementary Table 2. A significant difference in the NPAR was observed between survivors and nonsurvivors (*P* = 0.001) (Supplementary Fig. 3).

Univariable regression analysis (Crude model 1) revealed that the crude OR for in-hospital mortality, without adjusting for any covariates, was 4.58 (95% CI 1.86–11.27) in the high-NPAR group compared with the reference group with a low NPAR (*P* < 0.001). In addition, a statistically significant association was found between the NPAR (per one SD increase) and the risk of in-hospital mortality [OR, 1.93 (95% CI 1.23–3.04); *P* = 0.004] (Table 2 and Supplementary Table 3). Multivariable logistic regression (Full model 4) revealed that each one-SD increase in the NPAR was associated with a 1.85-fold increase in the risk of in-hospital mortality [OR, 2.85 (95% CI 1.35–6.03); *P* = 0.006]. Compared with the low NPAR group, the high NPAR group also had a significantly increased risk of in-hospital mortality, with an OR of 6.39 (95% CI 1.61–25.34; *P* = 0.008). The main regression results are summarized in Table 2. Furthermore, the multivariable-adjusted RCS model supported these findings, indicating that higher NPAR values were associated with increased in-hospital mortality (*P*_nonlinear_ = 0.84) (Supplementary Fig. 4).

**Table 2:** Association of neutrophil percentage-to-albumin ratio with in-hospital mortality among pediatric patients with acute fulminant myocarditis undergoing veno-arterial extracorporeal membrane oxygenation

NPAR	Crude model 1		Adjusted model 2		Adjusted model 3		Full model 4	
	OR (95% CI)	*P* value	OR (95% CI)	*P* value	OR (95% CI)	*P* value	OR (95% CI)	*P* value
Low NPAR	1.00		1.00		1.00		1.00	
High NPAR	4.58 (1.86–11.27)	** < 0.001**	4.83 (1.90–12.32)	** < 0.001**	6.73 (2.28–19.86)	** < 0.001**	6.39 (1.61–25.34)	**0.008**
NPAR per SD	1.93 (1.23–3.04)	**0.004**	2.19 (1.36–3.53)	**0.001**	2.50 (1.46–4.28)	** < 0.001**	2.85 (1.35–6.03)	**0.006**

Model 2, adjusted age, gender, weight; Model 3, adjusted model 2 + CPR, ECMO duration, MAP; Model 4, adjusted model 3 + Hct, Cr, pH, PaCO_2_, Lac, NLR.

Bold values indacate statistical significance

*CI* confidence interval, *OR* odds ratio, *CPR* cardiopulmonary resuscitation, *Cr* creatinine, *ECMO* extracorporeal membrane oxygenation, *Hct* hematocrit, *Lac* lactate level, *MAP* mean arterial pressure, *NLR* neutrophil–lymphocyte ratio, *NPAR* neutrophil percentage-to-albumin ratio, *PaCO*_*2*_ partial pressure of carbon dioxide, *SD* standard deviation

### Subgroup analyses

**Fig. 2:**
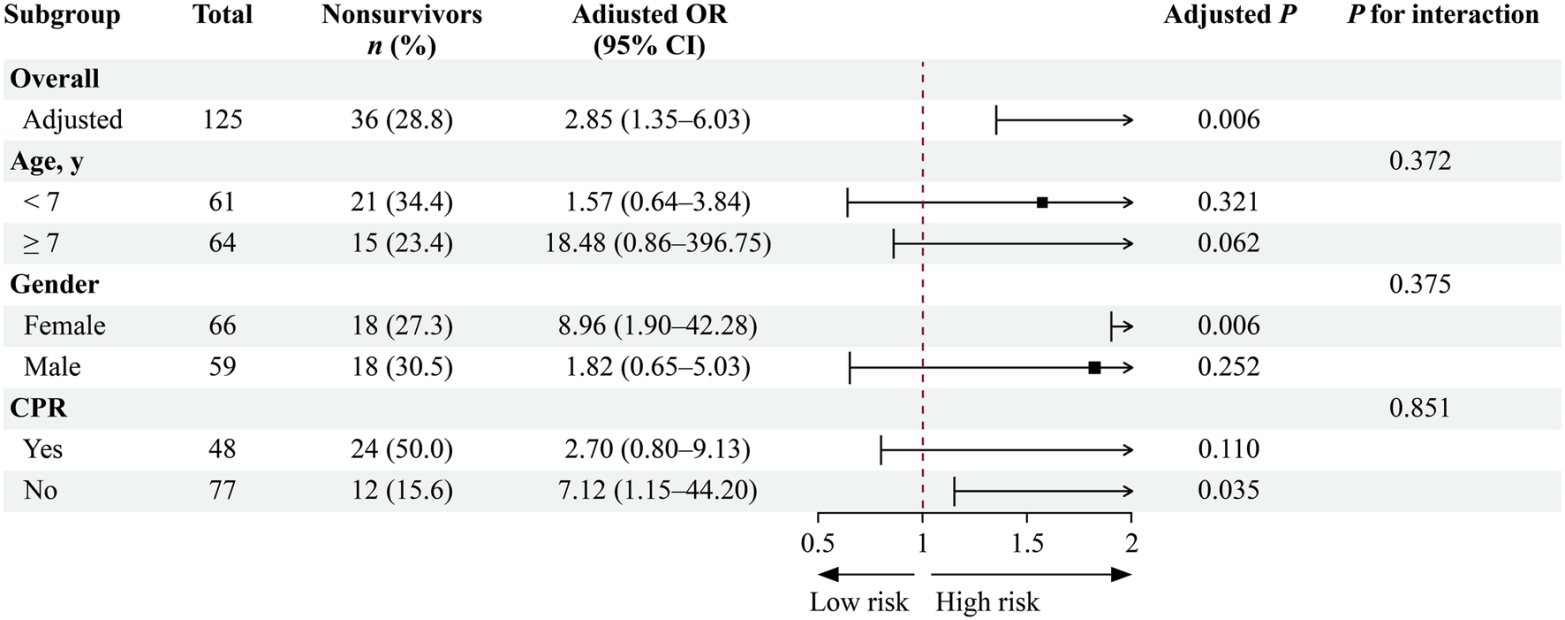
Subgroup analysis of the associations between the NPAR (per one SD) and VA-ECMO in-hospital mortality. *CPR* cardiopulmonary resuscitation, *CI* confidence interval, *NPAR* neutrophil percentage-to-albumin ratio, *OR* odds ratio, *SD* standard deviation, *VA-ECMO* veno-arterial extracorporeal membrane oxygenation

Subgroup analyses were conducted to explore the interactions between the NPAR and subgroup variables. The results are presented in Fig. 2. No significant interactions were observed between age (*P*_interaction_ = 0.372), sex (*P*_interaction_ = 0.375), CPR (*P*_interaction_ = 0.851), and NPAR.

### Predictive variables screening

**Fig. 3:**
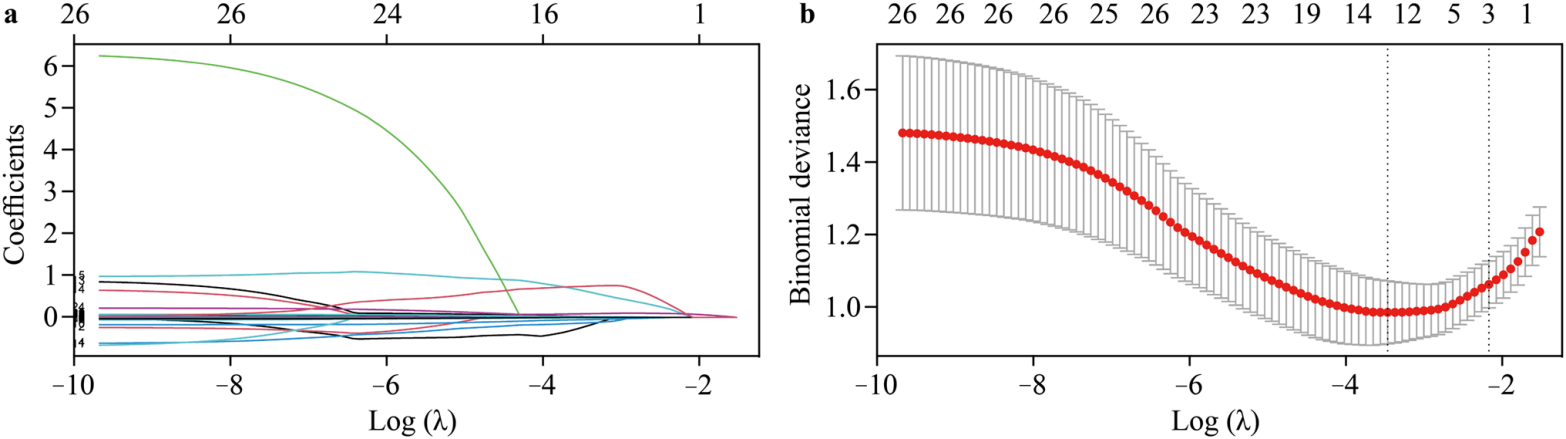
Predictive factor selection via least absolute shrinkage and selection operator (LASSO) regression with tenfold cross-validation. **a** LASSO coefficient profile, where each colored curve represents the trajectory of the coefficients for each variable. **b** Cross-validation curve for selecting the optimal value of the parameter λ. The left vertical dashed line indicates the parameter with the smallest error, while the right vertical dashed line corresponds to the parameter within one standard error (SE) of the minimum error. To avoid overfitting and simplify the model, three variables were retained based on the right line

A heatmap was generated to illustrate the correlations between continuous variables among all candidate variables (Supplementary Fig. 5). To minimize the risk of overfitting in the multivariable model, LASSO regression was applied to a set of 27 variables prior to further analysis. This step identified three nonzero parameter variables with potential predictive value for in-hospital mortality (Fig. 3). Additional screening of these three variables, along with factors (age, weight and sex) associated with the outcome demonstrated in previous studies, was performed via multivariable logistic regression analysis. This led to the final identification of four predictive factors associated with the risk of in-hospital mortality (Table 3).

**Table 3:** Multivariable logistic regression analysis to identify predictive factors

Variables	Univariable analysis			Multivariable analysis			
	Beta	OR (95% CI)	*P* value	Beta		OR (95% CI)	*P* value
Age							
< 7		1.00 (Reference)				1.00 (Reference)	
≥ 7	− 0.54	0.58 (0.27–1.28)	0.177	− 0.79		0.45 (0.17–1.19)	0.108
Weight	− 0.01	0.99 (0.96–1.02)	0.347				
Gender							
Female		1.00 (Reference)					
Male	1.69	1.17 (0.54–2.54)	0.690				
CPR							
No		1.00 (Reference)				1.00 (Reference)	
Yes	1.69	5.42 (2.35–12.50)	< 0.001	1.09		2.97 (1.07–8.23)	0.037
Lac	0.20	1.22 (1.12–1.32)	< 0.001	0.16		1.17 (1.07–1.29)	< 0.001
NPAR							
Low NPAR							
High NPAR	1.52	4.58 (1.86–11.27)	< 0.001	1.53		4.60 (1.56–13.62)	0.006

*CI* confidence interval, *OR* odds ratio, *CPR* indicates cardiopulmonary resuscitation, *Lac* lactate level, *NPAR* neutrophil percentage-to-albumin ratio

### Nomogram development and validation

**Fig. 4:**
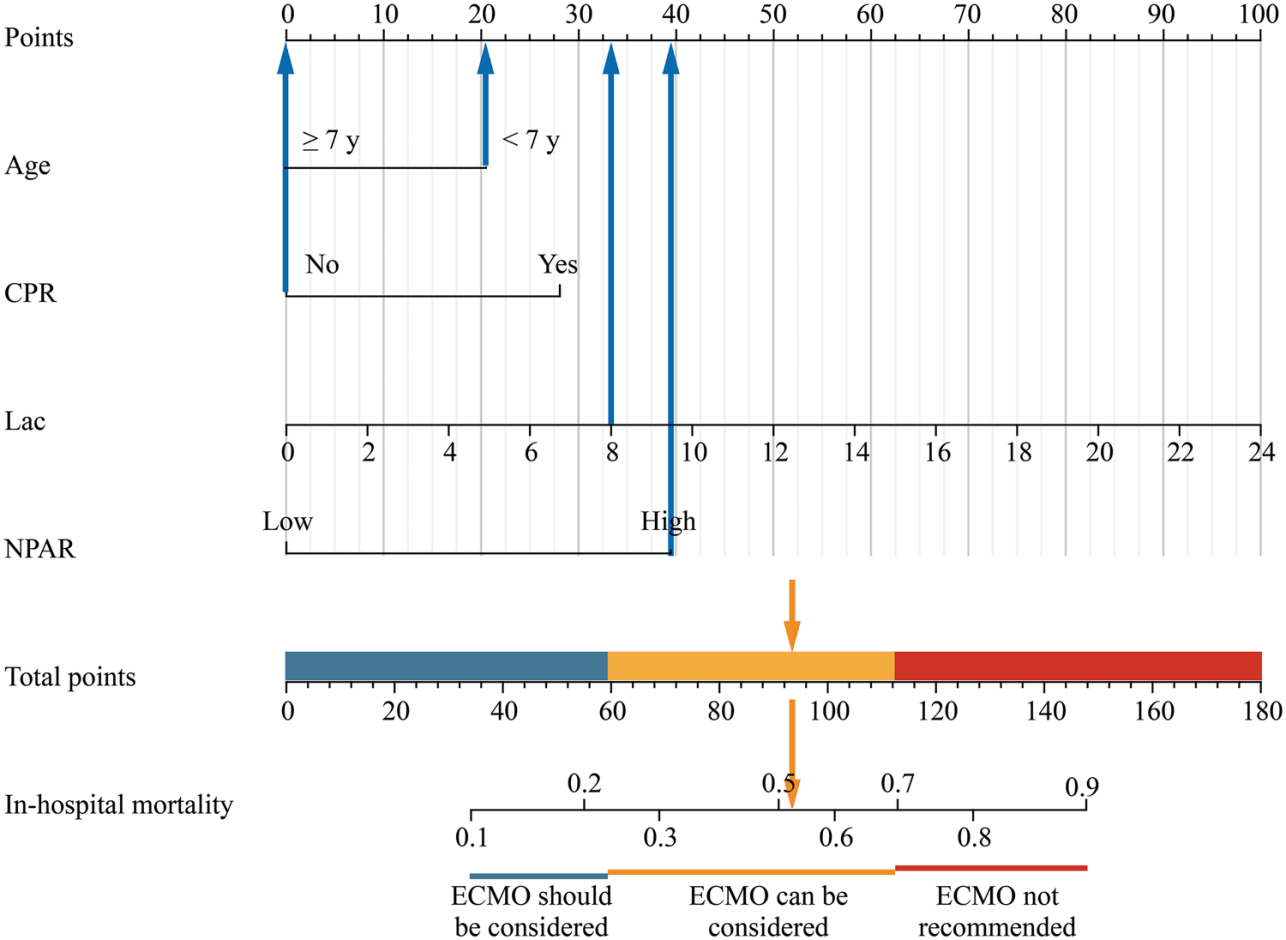
Nomogram for the prediction of in-hospital mortality after VA-ECMO. Low represents NPAR < 2.5; high represents NPAR ≥ 2.5. *CPR* cardiopulmonary resuscitation, *NPAR* neutrophil percentage-to-albumin ratio, *Lac* lactate level, *VA-ECMO* veno-arterial extracorporeal membrane oxygenation

We developed a nomogram model based on these four independent risk factors (Fig. 4). For each patient, the scores corresponding to the values obtained for each predictor were summed to yield a total score, with a higher total score indicating a greater risk of VA-ECMO in-hospital mortality. Furthermore, patients were divided into a low-risk group (score ≤ 59), an intermediate-risk group (59 < score ≤ 113), and a high-risk group (score ≥ 113) (*P* < 0.05). To further facilitate clinical application, we also created a web-based dynamic nomogram (https://ecmopredictionmodel.shinyapps.io/PeaceModel/. Supplementary Fig. 6).

**Fig. 5:**
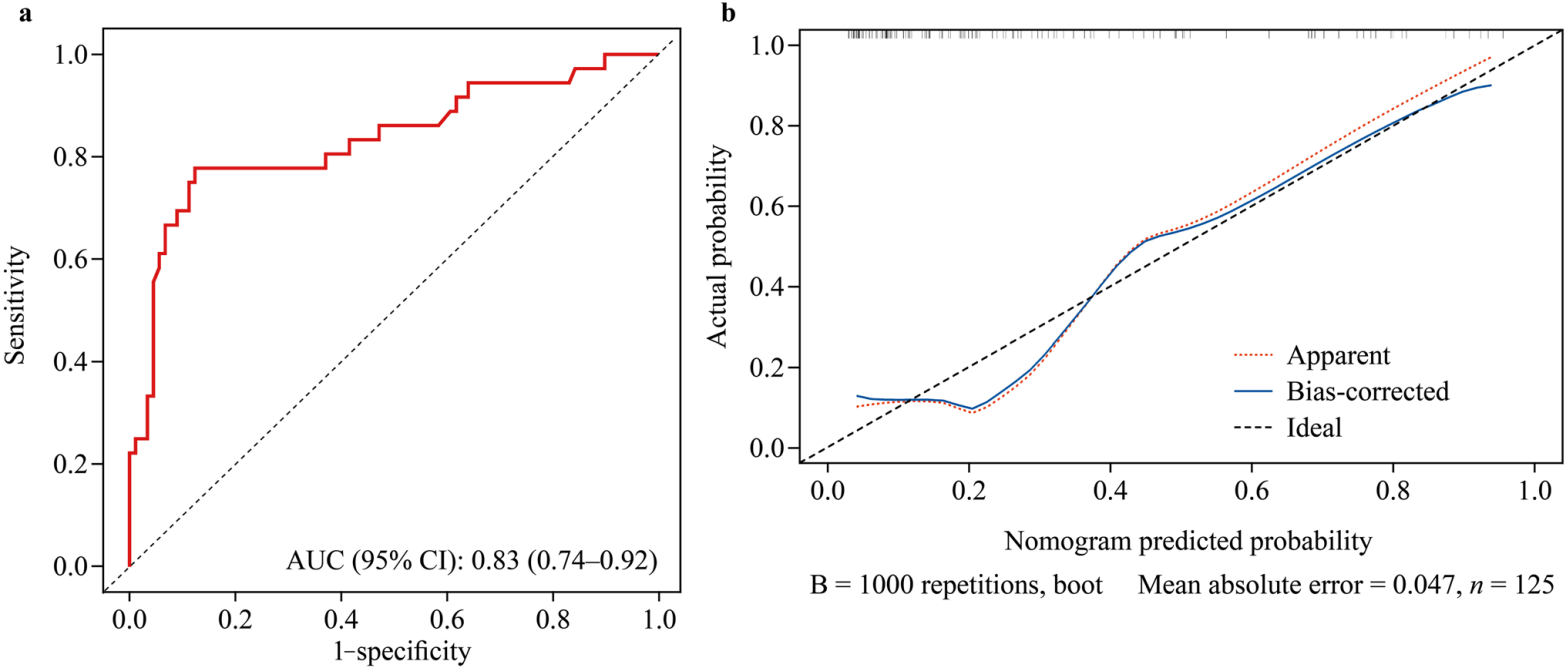
Receiver operating characteristic (ROC) curves and calibration curves of the nomogram prediction. **a** Discrimination assessment of the nomogram. **b** Accuracy assessment of the nomogram

**Fig. 6:**
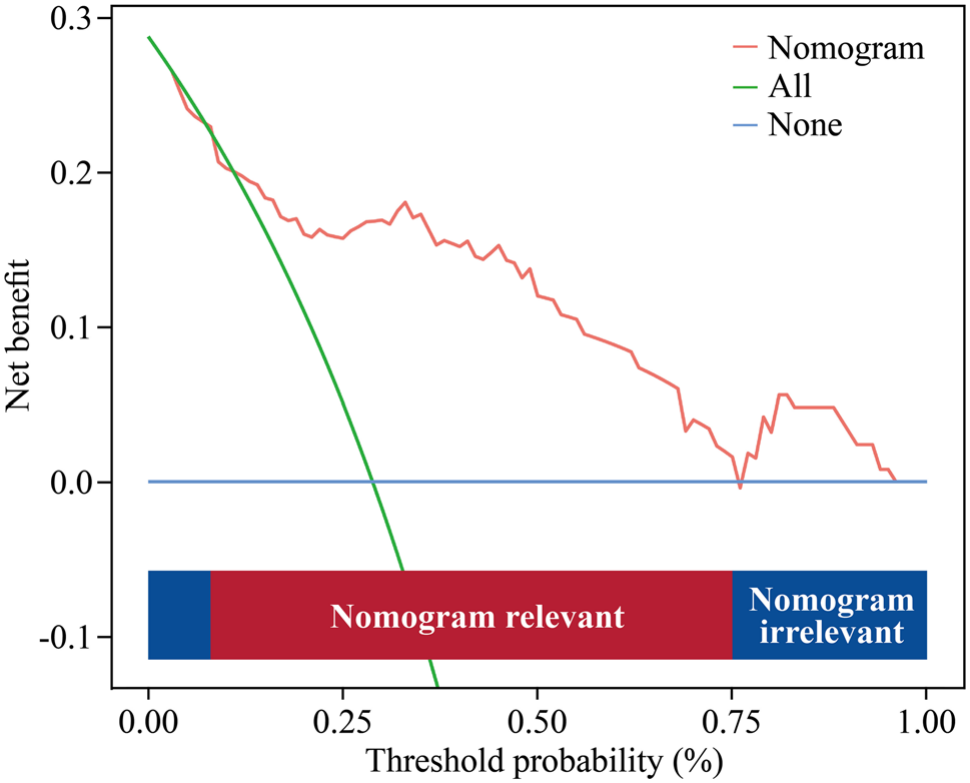
Decision curve analysis (DCA) of the nomogram prediction. The red line represents the predictive nomogram. The decision curve indicates that when the threshold is between about 8% and 75%, the application of this predictive model provides a net benefit compared to both the treat-all (green line) and treat-none (blue line) strategies

The discriminative power of the nomogram model was evaluated by receiver operating characteristic (ROC) curve analysis (Fig. 5a), with an area under the curve (AUC) of 0.833 (95% CI 0.74–0.92). The calibration curve (Fig. 5b) showed good agreement between the predicted and ideal models, with a mean absolute error of 0.047. The goodness of fit was further assessed via the Hosmer–Lemeshow test (*P* = 0.20). Internal validation by bootstrapping revealed that the model had an accuracy of 80.4%. DCA was conducted to evaluate the clinical applicability of the prediction model by examining the net benefit and threshold probability. The results showed that the model provided significant net clinical benefit for threshold probabilities ranging from 0.08 to 0.75 (Fig. 6).

To further evaluate the predictive role of the NPAR, we compared the performance of the nomogram with and without the NPAR using the NRI and IDI. The net gain in the NRI was 0.56 (*P* < 0.01), whereas the estimated value was 0.063 (*P* = 0.011) for the IDI (Supplementary Table 4).

## Discussion

AFM is typically characterized by the sudden onset of congestive heart failure or severe cardiogenic shock [2]. While VA-ECMO has become an effective treatment for pediatric patients with AFM, in-hospital mortality rates remain high [29]. To date, although several scoring systems [30–35] have been developed for pediatric ECMO patients, there is no one specifically for AFM to assess the prognosis of patients receiving VA-ECMO.

In the present study, we identified the NPAR as a significant risk factor for in-hospital mortality in AFM patients undergoing VA-ECMO. By incorporating the NPAR alongside other established risk factors, such as age, CPR and lactate level, we developed the a nomogram model (PEACE model) for predicting VA-ECMO in-hospital mortality in pediatric patients with AFM. To facilitate clinical utility, we also created a dynamic, web-based calculator. Moreover, compared with the model without the NPAR, the PEACE model displayed superior predictive performance. Our results underscore the potential of the NPAR as a valuable tool in clinical assessment to improve decision-making and patient management during VA-ECMO treatment.

Systemic inflammation plays a crucial role in the complex pathophysiology of critical illness [11, 36]. AFM is recognized as an inflammatory disease of the myocardium that can result from both infectious and noninfectious causes [2]. While the precise, comprehensive and systematic pathophysiological mechanisms of AFM remain unclear, it is widely accepted that an imbalanced immune response is central to its onset and progression [3]. Endomyocardial biopsy evidence reveals the presence of immune cell infiltration (such as neutrophils, monocytes, and lymphocytes) in the necrotic myocardium [37]. Furthermore, VA-ECMO support can exacerbate inflammation through direct contact between circulating blood and the extracorporeal circuit. This triggers complement activation, cytokine release, and leukocyte infiltration [38–41], leading to further neutrophil activation and tissue damage [11].

Neutrophils, as key mediators of innate immunity, rapidly increase in number in response to systemic inflammation and are often elevated at the initiation of ECMO [42–44]. However, in this study, neutrophil count alone did not show a statistically significant difference between survivor and non-survivor groups. In contrast, serum albumin levels exhibited a more pronounced divergence. Albumin, a negative acute-phase reactant, plays several critical roles in severe illness, including maintaining oncotic pressure, modulating immune responses, and exerting antioxidant effects [45–48]. During systemic inflammation, albumin levels decrease due to increased vascular permeability, hepatic reprioritization, and increased catabolism. Furthermore, during ECMO, factors such as hemodilution and protein loss through the extracorporeal membrane contribute to hypoalbuminemia [49]. Previous studies have demonstrated that hypoalbuminemia during ECMO is independently associated with increased mortality [49]. The NPAR integrates these two parameters and offers a more comprehensive index reflecting both inflammation and nutritional/metabolic status. In various critical care settings, an elevated NPAR has been independently associated with increased mortality, including in patients with ST-elevation myocardial infarction [50] and cardiogenic shock [51]. The strength of the NPAR lies in its ability to amplify the prognostic signal of hypoalbuminemia by incorporating neutrophil levels, particularly in cases where these parameters do not deviate significantly from the normal range, an aspect that is often overlooked by clinicians. Thus, the NPAR may serve as a more stable and sensitive marker than either parameter alone. In this study, the inclusion of the NPAR in the nomogram significantly strengthened the prognostic performance over models without it. The potential mechanisms underlying the association between an elevated NPAR and poor prognosis include neutrophil-driven tissue injury, immune dysregulation, and albumin depletion secondary to systemic inflammation and ECMO-induced capillary leakage. Collectively, these mechanisms underscore the biological plausibility of the NPAR as a valuable prognostic biomarker. Nevertheless, further comprehensive research is needed to elucidate the exact underlying mechanism involved.

In clinical practice, younger age is recognized as an independent risk factor for in-hospital mortality in pediatric patients receiving VA-ECMO support [52]. We found similar results in this study, which are consistent with the results of a previous meta-analysis [53]. This may be because younger children are more likely to develop neurological complications during VA-ECMO support [54], resulting in higher mortality. For patients undergoing CPR, the condition itself is very serious, and cardiac arrest leads to ischemia and hypoxia of organs and tissues throughout the body. If cardiac function cannot be restored in time, the patient is vulnerable to brain damage and multiple organ dysfunction, resulting in an increased risk of death [55]. In addition, the restoration of blood flow after CPR can cause ischemia‒reperfusion injury, leading to cellular damage and inflammatory responses. These injuries and inflammatory responses may exacerbate damage to other organs and tissues, thereby affecting patient prognosis. Elevated lactate levels indicate circulatory disturbances and inadequate tissue perfusion [56, 57], which may indicate damage to multiple organ functions, thereby affecting the body's metabolic processes and exacerbating lactic acid accumulation. In addition, once VA-ECMO is initiated, the body's inflammatory response and immune system lead to the release of inflammatory mediators that interfere with the body's cellular respiratory function, thereby increasing the risk of death.

Age, CPR, lactate level, and the NPAR can be easily obtained from patients receiving VA-ECMO. On the basis of these four variables, we constructed a PEACE model to predict in-hospital mortality, specifically in children with AFM supported by VA-ECMO. In comparison, previous risk stratification tools, such as the Pediatric Extracorporeal Membrane Oxygenation Prediction (PEP) [30] model and the Pediatric Survival After Veno-Arterial ECMO (Pedi-SAVE) score [34], were derived from broader pediatric ECMO populations. The PEP model, developed using international multicenter registry data, includes a wide range of demographic, clinical, and laboratory variables and has an AUC of 0.75 for predicting in-hospital mortality in pediatric patients receiving ECMO for any indication. The Pedi-SAVE score, on the other hand, was designed for patients receiving VA-ECMO and includes both precannulation and postcannulation scores. The precannulation score yielded an AUC of 0.64, and the postcannulation score yielded an AUC of 0.71. Unlike these models, the PEACE model is tailored to a specific and clinically distinct subgroup—pediatric patients with AFM, a condition characterized by rapid cardiovascular collapse. Our model demonstrated superior prognostic performance, with an AUC of 0.83, indicating better discriminative ability than the PEP and Pedi-SAVE scores. In addition, the use of only four objective and commonly available variables enhances its practicality for early bedside application. Furthermore, internal validation via tenfold cross-validation confirmed the robustness and stability of the PEACE model. These results suggest that disease-specific models may outperform general ECMO prediction tools in certain contexts and highlight the need for further prospective multicenter validation.

Notably, the NPAR is derived from two readily available laboratory parameters—neutrophil count and serum albumin—which are routinely tested in most clinical settings, including those with limited access to advanced diagnostic tools. This makes the NPAR a cost-effective and easily applicable marker for early risk stratification in pediatric patients with AFM undergoing VA-ECMO. Its inclusion in the PEACE model adds to the model’s practicality and potential for broader implementation, particularly in resource-constrained environments.

Predictive models are commonly used to select appropriate patients for specific treatments [57, 58]. However, for pediatric patients with AFM who require VA-ECMO support, clinicians continue to face significant challenges in decision-making, even with the assistance of predictive models. Such models can offer valuable prognostic information to both families and clinicians regarding the expected outcomes of VA-ECMO in patients with similar risk conditions while also helping clinicians identify high-risk pediatric patients and providing a theoretical foundation for clinical decision making.

This study has several limitations. The study included only pediatric patients with AFM receiving VA-ECMO support, which ensures that the predictive model has a well-defined target population and improves its accuracy. However, several limitations must be considered. First, this study was a retrospective observational study with a small sample size, which inevitably is prone to selection bias, limiting the generalizability of its predictive accuracy. Second, some data had a high proportion of missing data, and variables that could influence the study, such as LVEF, were not included in the analysis. This is due to the lack of standardization in echocardiographic assessments across participating centers, leading to significant variations in measurement timing, techniques, and reporting. Third, the model was built on the basis of patients who had undergone VA-ECMO, so bias may have been introduced when the prognostic probability of VA-ECMO in children with AFM who had not received VA-ECMO was assessed. Finally, the model was only internally validated within the current cohort. The absence of external validation limits its generalizability to other settings and populations. Future studies should focus on conducting prospective, multicenter validation using independent cohorts to confirm the predictive value of the PEACE model and the NPAR across diverse clinical settings.

In conclusion, this study suggests that the NPAR is a potentially valuable independent predictor of in-hospital mortality in pediatric patients with AFM receiving VA-ECMO. Elevated NPAR levels were strongly associated with an increased risk of in-hospital mortality. The development of a predictive model incorporating the NPAR together with age, CPR and lactate level could enhance the ability to predict the prognosis of pediatric patients supported by VA-ECMO. These findings highlight that monitoring the NPAR and applying a predictive model could help clinicians select patients who are more likely to benefit from VA-ECMO.

## Supplementary Information

Below is the link to the electronic supplementary material.

Supplementary file1 (PDF 1157 KB)

## Data Availability

The data of this study are available from the corresponding author upon reasonable request.
