# Supplementary material for: Predictive role of neutrophil percentage-to-albumin ratio in acute fulminant myocarditis patients receiving extracorporeal membrane oxygenation
Source: World J Pediatr. 2025 Jul 17;21(8):823–35. doi: 10.1007/s12519-025-00940-4 (PMC12380884; doi:10.1007/s12519-025-00940-4)

Predictive role of neutrophil percentage-to-albumin ratio in acute fulminant myocarditis patients receiving extracorporeal membrane oxygenation

Supplementary Appendix

Content

TRIPOD Checklist 2

Table 1: The detailed information of collected and defined variables..................................3

Table 2: Baseline characteristics of study population according to NPAR cutoff value 4

Table 3: The univariate regression analysis for in-hospital mortality in pediatric patients with AFM receiving VA-ECMO 6

Table 4: Comparisons of the nomogram with the model without NPAR 7

Fig. 1: The distribution of missing data and the multiple imputation data. 8

Fig. 2: The cutoff value of NPAR obtained through receiver operating characteristic curve analysis...9

Fig. 3: The violin plot illustrates the distribution of NPAR levels among survivors and nonsurvivors.. 10

Fig. 4: Multivariate adjusted RCS plots to assess the dose-response relationship between NPAR and in-hospital mortality..11

Fig. 5: The heatmap illustrates the correlations between continuous variables among all independent variables 12

Fig. 6: The web-based dynamic nomogram 13

TRIPOD Checklist

Section/Topic	Item		Checklist Item	Page
**Title and abstract**				
Title	1	D;V	Identify the study as developing and/or validating a multivariable prediction model, the target population, and the outcome to be predicted.	1
Abstract	2	D;V	Provide a summary of objectives, study design, setting, participants, sample size, predictors, outcome, statistical analysis, results, and conclusions.	2
**Introduction**				
Background and objectives	3a	D;V	Explain the medical context (including whether diagnostic or prognostic) and rationale for developing or validating the multivariable prediction model, including references to existing models.	5
	3b	D;V	Specify the objectives, including whether the study describes the development or validation of the model or both.	6
**Methods**				
Source of data	4a	D;V	Describe the study design or source of data (e.g., randomized trial, cohort, or registry data), separately for the development and validation data sets, if applicable.	6
	4b	D;V	Specify the key study dates, including start of accrual; end of accrual; and, if applicable, end of follow-up.	6
Participants	5a	D;V	Specify key elements of the study setting (e.g., primary care, secondary care, general population) including number and location of centres.	6
	5b	D;V	Describe eligibility criteria for participants.	6
	5c	D;V	Give details of treatments received, if relevant.	6-7
Outcome	6a	D;V	Clearly define the outcome that is predicted by the prediction model, including how and when assessed.	8
	6b	D;V	Report any actions to blind assessment of the outcome to be predicted.	-
Predictors	7a	D;V	Clearly define all predictors used in developing or validating the multivariable prediction model, including how and when they were measured.	8 Supplementary Table 1
	7b	D;V	Report any actions to blind assessment of predictors for the outcome and other predictors.	-
Sample size	8	D;V	Explain how the study size was arrived at.	-
Missing data	9	D;V	Describe how missing data were handled (e.g., complete-case analysis, single imputation, multiple imputation) with details of any imputation method.	10
Statistical analysis methods	10a	D	Describe how predictors were handled in the analyses.	9-10
	10b	D	Specify type of model, all model-building procedures (including any predictor selection), and method for internal validation.	9-10
	10c	V	For validation, describe how the predictions were calculated.	-
	10d	D;V	Specify all measures used to assess model performance and, if relevant, to compare multiple models.	10
	10e	V	Describe any model updating (e.g., recalibration) arising from the validation, if done.	-
Risk groups	11	D;V	Provide details on how risk groups were created, if done.	9-10
Development vs. validation	12	V	For validation, identify any differences from the development data in setting, eligibility criteria, outcome, and predictors.	-
**Results**				
Participants	13a	D;V	Describe the flow of participants through the study, including the number of participants with and without the outcome and, if applicable, a summary of the follow-up time. A diagram may be helpful.	10-11 Supplementary Fig. 1
	13b	D;V	Describe the characteristics of the participants (basic demographics, clinical features, available predictors), including the number of participants with missing data for predictors and outcome.	11 Table 1
Model development	14a	D	Specify the number of participants and outcome events in each analysis.	11
	14b	D	If done, report the unadjusted association between each candidate predictor and outcome.	11 Supplementary Table 3
Model specification	15a	D	Present the full prediction model to allow predictions for individuals (i.e., all regression coefficients, and model intercept or baseline survival at a given time point).	12-13
	15b	D	Explain how to the use the prediction model.	12-13
Model performance	16	D;V	Report performance measures (with CIs) for the prediction model.	13
Model-updating	17	V	If done, report the results from any model updating (i.e., model specification, model performance).	-
**Discussion**				
Limitations	18	D;V	Discuss any limitations of the study (such as nonrepresentative sample, few events per predictor, missing data).	17
Interpretation	19a	V	For validation, discuss the results with reference to performance in the development data, and any other validation data.	-
	19b	D;V	Give an overall interpretation of the results, considering objectives, limitations, results from similar studies, and other relevant evidence.	14-17
Implications	20	D;V	Discuss the potential clinical use of the model and implications for future research.	17
**Other information**				
Supplementary information	21	D;V	Provide information about the availability of supplementary resources, such as study protocol, Web calculator, and data sets.	18
Funding	22	D;V	Give the source of funding and the role of the funders for the present study.	Title page

Table 1. The detailed information of collected and defined variables.

**Demographic information***	age, weight, gender
**Routine laboratory tests †**	hemoglobin (HB), red blood cell distribution width (RDW), hematocrit (Hct), white blood cell (WBC), neutrophil count, neutrophil percentage, lymphocyte count, Platelet (PLT), C-reactive protein (CRP), albumin, creatinine (Cr), cardiac troponin I (cTNI), brain natriuretic peptide (BNP), pH, partial pressure of oxygen (PaO_2), partial pressure of carbon dioxide ($PaCO_2$), lactate levels (Lac), left ventricular ejection fraction (LVEF)
**Clinical characteristics †**	systolic blood pressure (SBP), diastolic blood pressure (DBP), and mean arterial pressure (MAP)
**NLR †**	the ratio of neutrophil count to lymphocyte count
**NPAR †**	the ratio of neutrophil percentage to albumin
**Others**	duration of VA-ECMO support, cardiopulmonary resuscitation (CPR)

*, variables were recorded upon admission.

†, variables were recorded at 12 hours after VA-ECMO initiation.

Table 2. Baseline characteristics of study population according to NPAR cutoff value.

Characteristics	Total	Low NPAR (<2.5)	High NPAR (≥ 2.5)	*P* value
Number	125	95	30	
**Demographics**				
Age, year	7 (4.17, 10)	7 (4, 10)	7 (4.88, 10.21)	0.719
Gender, n (%)				0.23
Male	59 (47.20)	43 (43.88)	16 (59.26)	
Weight, kg	23.1 (15.78, 36)	22 (16, 35)	30 (15.39, 39)	0.331
**Clinical characteristics**				
CPR, n (%)	48 (38.40)	35 (35.71)	13 (48.15)	0.341
ECMO duration, hour	120.17 (92, 168)	133.71 (96.02, 179.68)	107 (69.48, 147.5)	0.025
LOHS, day	22.76 ± 14.12	23.51 ± 12.56	20.02 ± 18.77	0.368
Blood pressure, mmHg				
Systolic	76.0 (60.0, 95.0)	77.0 (60.0, 95.75)	73.0(59.3, 89.0)	0.674
Diastolic	47.0 (35.0, 60.0)	47.0 (35.0, 60.0)	51.0 (36.0, 63.5)	0.67
Mean arterial pressure	56.67 (44.33, 70.33)	56.67 (43.58, 70.25)	54 (45.83, 71)	0.909
**Laboratory tests**				
Hemoglobin, g/L	113.0 (98.0, 124.0)	116.5 (103.0, 125.0)	99.0 (94.5, 111.0)	0.014
Red blood distribution width, %	14.0 (12.9, 35.2)	13.75 (12.72, 33.45)	15.4 (13.75, 43.5)	0.01
Hematocrit, %	32.57 ± 5.53	33.11 ± 5.7	30.61 ± 4.45	0.019
White blood cell, ×10^9/L	11.29 (8.06, 15.33)	10.48 (7.49, 13.46)	15.22 (10.3, 20.08)	0.004
Neutrophil count, ×10^9/L	7.99 (5.11, 11.6)	7.36 (4.91, 10.08)	11.6 (8.68, 16.2)	< 0.001
Neutrophil percentage, %	74.72 (64, 83.3)	73.7 (58.83, 79.68)	84.2 (74.25, 88.25)	< 0.001
Lymphocyte count, ×10^9/L	1.74 (1.03, 2.8)	1.77 (1.07, 2.63)	1.62 (0.9, 2.96)	0.886
Nenutrophil-lymphocyte ratio	4.44 (2.34, 8.45)	3.94 (2.06, 7.5)	6.36 (4.17, 12.94)	0.019
Platelet count,×10^9/L	205 (126, 280)	215.5 (141.75, 295.75)	134 (102.5, 219)	0.017
C-reactive protein, mg/L	5.68 (2.73, 13)	5.23 (2.7, 12)	9 (2.85, 19.39)	0.383
Albumin, g/L	35.5 (32.7, 39.46)	36.88 (34.7, 40.36)	29.4 (26.6, 32)	< 0.001
Creatinine, μmol/L	66 (48, 105.5)	62.03 (47.55, 92)	91 (53.75, 166.1)	0.005
Cardiac troponin I, ng/ml	4.26 (1.55, 10.05)	5.18 (1.68, 10.12)	3.19 (0.91, 8.66)	0.244
Brain natriuretic peptide, pg/ml	15000 (5542, 30790.2)	15000 (7962.95, 29750.65)	13963 (4800, 32599.6)	0.796

pH	7.33 (7.2, 7.43)	7.34 (7.2, 7.43)	7.33 (7.22, 7.43)	0.737
PaO_2, mmHg	91.5 (51.2, 168)	99.35 (47.3, 169.69)	85.9 (55.7, 152.35)	0.874
$PaCO_2$, mmHg	34 (28.2, 42.7)	35.85 (29.41, 43.7)	32.4 (24.6, 38.37)	0.191
Lactate, mmol/L	4.9 (2.4, 11.6)	4.4 (2.32, 10.63)	7.9 (2.6, 14.8)	0.139
Death	36 (28.80)	21 (21.43)	15 (55.56)	0.001

Data are presented as counts and proportions for categorical variables and as mean ± standard deviation (SD) or median (interquartile range, IQR) for continuous variables.

CPR indicates cardiopulmonary resuscitation; ECMO, extracorporeal membrane oxygenation; LOHS, length of hospital stay; NPAR, neutrophil percentage to albumin ratio; PaO_2, partial pressure of oxygen; and $PaCO_2$, partial pressure of carbon dioxide.

Table 3. The univariate regression analysis for in-hospital mortality in pediatric patients with AFM receiving VA-ECMO.

Variables	β	S.E	Z	*P*	OR (95%CI)
Age ≥7	-0.54	0.40	-1.35	0.177	0.58 (0.27 - 1.28)
Male	0.16	0.40	0.40	0.690	1.17 (0.54 - 2.54)
CPR	1.69	0.43	3.96	<.001	5.42 (2.35 - 12.50)
Weight	-0.01	0.01	-0.94	0.347	0.99 (0.96 - 1.02)
ECMO duration	0.00	0.00	0.37	0.713	1.00 (1.00 - 1.00)
Blood pressure					
Systolic	-0.02	0.01	-2.59	0.010	0.98 (0.97 - 0.99)
Diastolic	-0.03	0.01	-2.63	0.009	0.97 (0.96 - 0.99)
Mean arterial pressure	-0.02	0.01	-2.68	0.007	0.98 (0.96 - 0.99)
Hemoglobin	-0.02	0.01	-2.01	0.044	0.98 (0.96 - 0.99)
Red blood distribution width	0.04	0.01	2.51	0.012	1.04 (1.01 - 1.07)
Hematocrit	-0.12	0.04	-3.03	0.002	0.89 (0.82 - 0.96)
White blood cell	0.06	0.03	2.07	0.039	1.07 (1.01 - 1.13)
Neutrophil count	0.07	0.04	2.00	0.045	1.07 (1.01 - 1.15)
Neutrophil percentage	-0.00	0.01	-0.14	0.888	1.00 (0.98 - 1.02)
Lymphocyte count	0.12	0.08	1.55	0.122	1.13 (0.97 - 1.32)
Platelet count	-0.01	0.00	-2.04	0.041	0.99 (0.99 - 0.99)
C-reactive protein	-0.01	0.01	-0.90	0.371	0.99 (0.96 - 1.01)
Albumin	-0.10	0.03	-3.08	0.002	0.90 (0.84 - 0.96)
Creatinine	0.01	0.00	3.28	0.001	1.01 (1.01 - 1.02)
Cardiac troponin I	0.04	0.02	2.03	0.042	1.04 (1.01 - 1.08)
Brain natriuretic peptide	0.00	0.00	1.25	0.211	1.00 (1.00 - 1.00)
pH	-3.24	1.13	-2.87	0.004	0.04 (0.00 - 0.36)
PaO2	-0.00	0.00	-1.26	0.207	1.00 (0.99 - 1.00)
PaCO2	0.03	0.01	2.62	0.009	1.03 (1.01 - 1.06)
Lactate	0.20	0.04	4.83	<.001	1.22 (1.13 - 1.32)
Neutrophil-lymphocyte ratio	-0.02	0.02	-1.13	0.258	0.98 (0.95 - 1.01)
High NPAR	1.52	0.46	3.32	<.001	4.58 (1.86 - 11.27)

AFM indicates acute fulminant myocarditis; CPR, cardiopulmonary resuscitation; ECMO, extracorporeal membrane oxygenation; LOHS, length of hospital stay; NPAR, neutrophil percentage to albumin ratio; PaO_2, partial pressure of oxygen; and $PaCO_2$, partial pressure of carbon dioxide; and VA-ECMO, veno-arterial extracorporeal membrane oxygenation.

Table 4. Comparisons of the nomogram with the model without NPAR.

Indicators	Nomogram	Model without NPAR	*P* value
**NRI**	0.56 (0.21-0.92)		0.0017
**IDI**	0.06 (0.01-0.11)		0.011

IDI indicates integrated discrimination improvement; NPAR, neutrophil percentage to albumin ratio; and NRI, net reclassification improvement.

Fig. 1 The distribution of missing data (A) and the multiple imputation data (B).

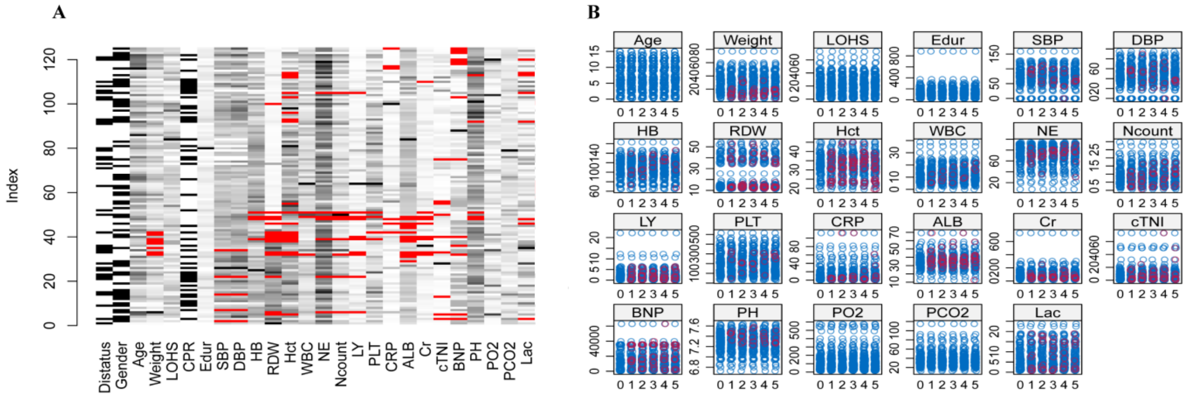

Fig. 2 The cutoff value of NPAR obtained through receiver operating characteristic curve analysis.

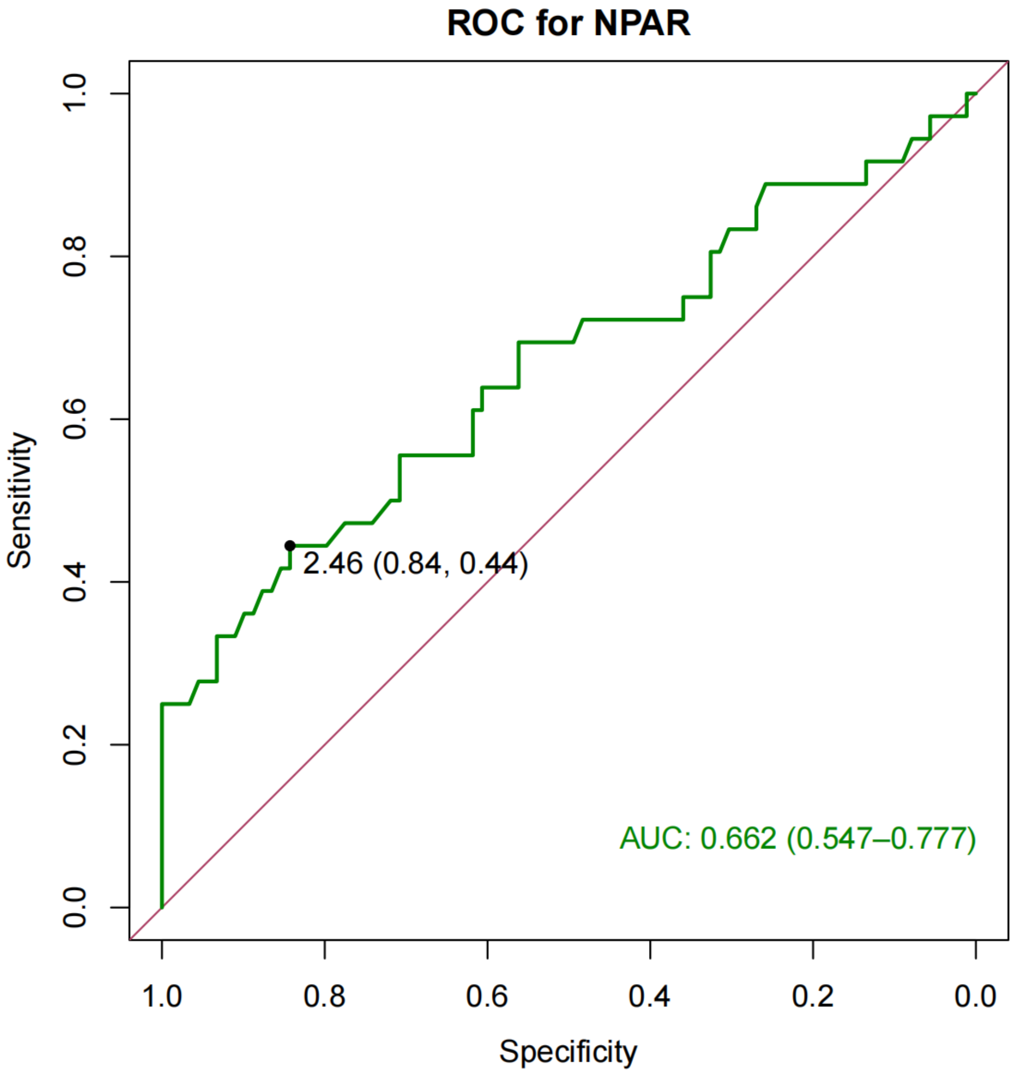

Fig. 3 The violin plot illustrates the distribution of NPAR levels among survivors and nonsurvivors.

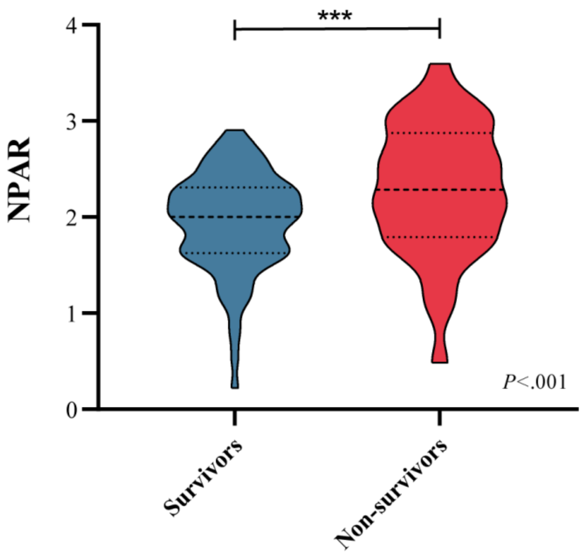

Figure S4 Multivariate adjusted RCS plots to assess the dose-response relationship between NPAR and in-hospital mortality.

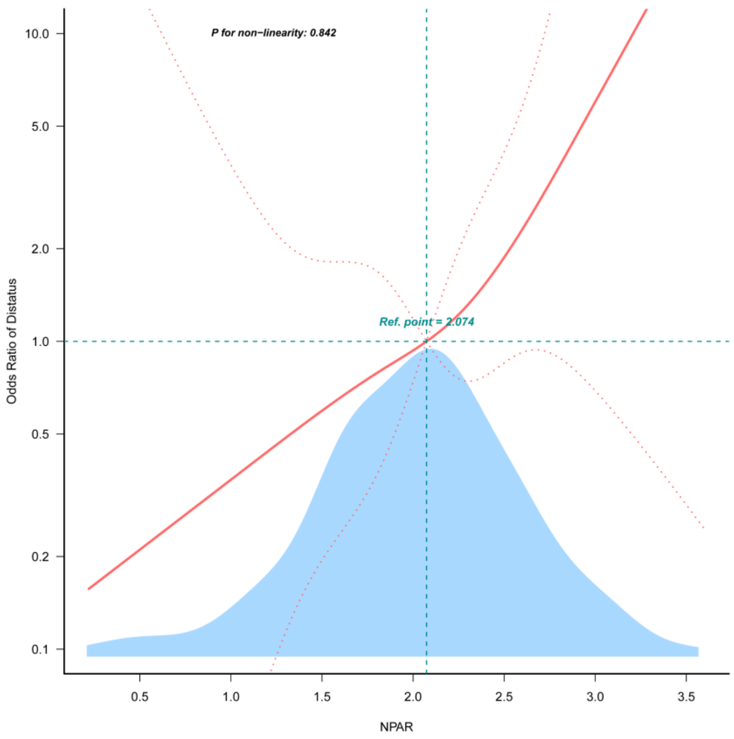

Fig. 5 The heatmap illustrates the correlations between continuous variables among all independent variables

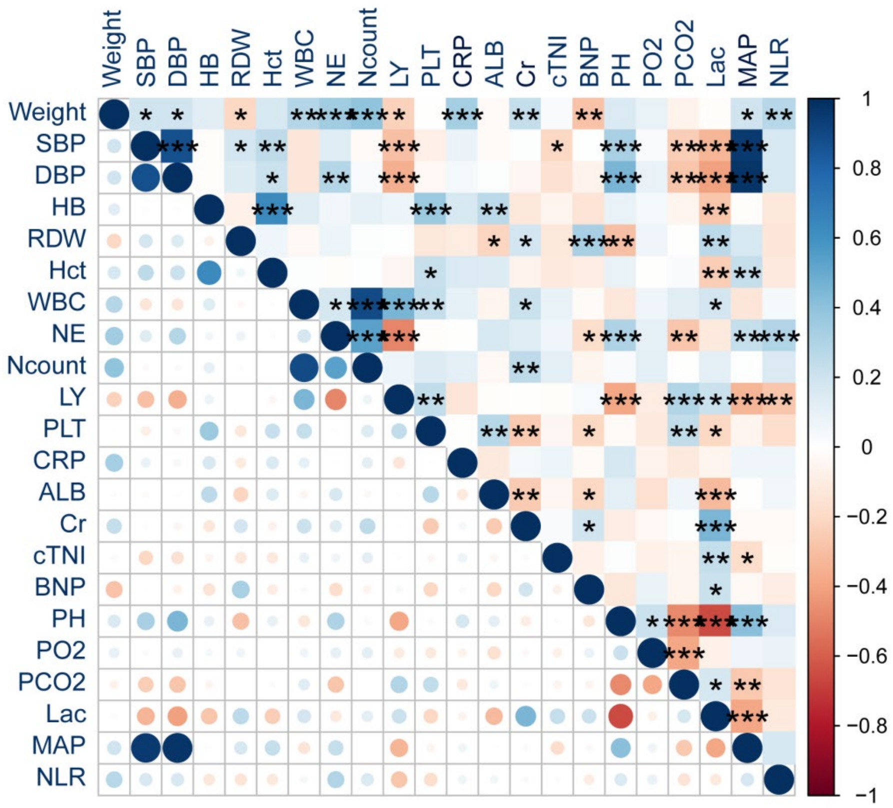

Fig. 6 The web-based dynamic nomogram.

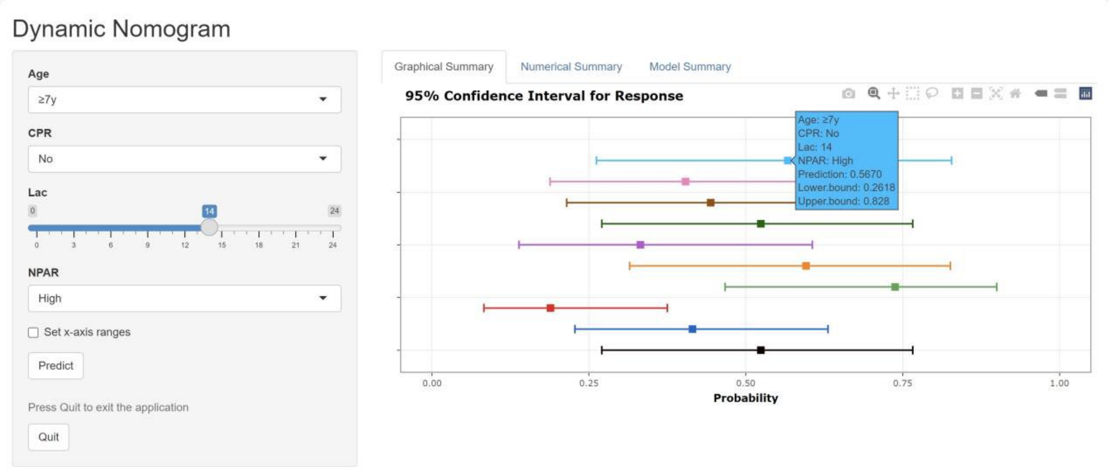

Supplement: Supplementary file 1 — Supplementary file1 (PDF 1157 KB) [file 12519_2025_940_MOESM1_ESM.pdf]
